# Family history of haematopoietic malignancies and non-Hodgkin's lymphoma risk in the California Teachers Study

**DOI:** 10.1038/sj.bjc.6604881

**Published:** 2009-01-20

**Authors:** Y Lu, J Sullivan-Halley, W Cozen, E T Chang, K Henderson, H Ma, D Deapen, C Clarke, P Reynolds, S L Neuhausen, H Anton-Culver, G Ursin, D West, L Bernstein

**Affiliations:** 1 Department of Preventive Medicine, USC/Norris Comprehensive Cancer Center NOR-3429, University of Southern California, 1441 Eastlake Avenue, Los Angeles, CA 90089-9175, USA; 2 Division of Cancer Etiology, Department of Population Sciences, City of Hope National Medical Center, 1500 East Duarte Rd, Duarte, CA 91010, USA; 3 Northern California Cancer Center, 2201 Walnut Avenue, Fremont, CA 94538, USA; 4 Department of Epidemiology, School of Medicine, University of California Irvine, Irvine, CA 92697-7550, USA; 5 Department of Nutrition, University of Oslo, Boks 1072 Blindern, NO-0316 Oslo, Norway

**Keywords:** non-Hodgkin's lymphoma, family history, haematopoietic malignancy, lymphoma, leukaemia

## Abstract

Family history of haematopoietic malignancies appears to be a risk factor for non-Hodgkin's lymphoma (NHL), but whether risk varies by family member's gender is unclear. Among 121 216 women participating in the prospective California Teachers Study, NHL risk varied by type of haematopoietic malignancy and gender of the relative.

Evidence from epidemiologic studies shows that the risk of non-Hodgkin's lymphoma (NHL) is increased by approximately twofold for individuals with a first-degree family history (parent, sibling or child) of haematopoietic malignancy (Pottern *et al*, 1991; Zhu *et al*, 1998; Chiu *et al*, 2004; Altieri *et al*, 2005; Chang *et al*, 2005; Goldin *et al*, 2005; Wang *et al*, 2007). However, the underlying mechanisms are poorly understood.

A recent analysis from the International Lymphoma Epidemiology (InterLymph) Consortium of NHL case–control studies found an increased risk of NHL for individuals who reported having a first-degree relative with a haematopoietic malignancy (NHL, Hodgkin's lymphoma, leukaemia or multiple myeloma) (Wang *et al*, 2007). They also found that NHL risk differed by the gender and familial relationship of the affected relative. These findings were based on case–control studies using retrospectively self-reported data on family history, and were therefore potentially subject to survival bias and differential recall bias (Chang *et al*, 2006). To understand the underlying disease mechanisms in NHL better, we used data from the prospective California Teachers Study to investigate NHL risks associated with first-degree family history.

## Materials and methods

A detailed description of the California Teachers Study has been published elsewhere (Bernstein *et al*, 2002). In this analysis, we included 121 216 women who were California residents, were under the age of 85 years at baseline and had no prior history of a haematopoietic malignancy. Use of human subjects in this study was approved by each participating institution.

Incident diagnoses of B-cell NHL (International Classification of Diseases for Oncology third edition morphology codes: 9590, 9591, 9670–9675, 9680–9699, 9727, 9823, 9832, 9835 and 9836) were identified through annual linkages with the population-based California Cancer Registry. Person-time was accrued from the date a participant completed her baseline questionnaire until the first occurrence of either a diagnosis of a B-cell NHL or a censoring event: a move outside of California; a first diagnosis of another haematopoietic malignancy not included in our case definition; death; or 31 December 2005.

The self-administered, baseline questionnaire collected detailed information on personal and family history of cancer. Having ‘no family history’ of a haematopoietic malignancy was defined as reporting no first-degree relative with NHL, Hodgkin's lymphoma or leukaemia.

We used Cox proportional hazards regression models, stratified by age in years at baseline, to estimate hazard rate ratios (RR) and 95% confidence intervals (CIs) for B-cell NHL, using age in days from baseline until the end of follow-up for the time under observation. We assessed potential confounders including race; history of a full-term pregnancy; baseline residential socioeconomic status, smoking status, height and body mass index (BMI); and alcohol consumption 1 year before baseline. We did not include any of these variables in our final statistical models as none changed the risk estimates by as much as 5%. All statistical analyses were performed using SAS version 9.1 (SAS Institute Inc.).

## Results

During an average of 9.3 years of follow-up, 478 California Teachers Study participants were diagnosed with B-cell NHL. Table 1 summarises the distribution of several characteristics according to family history of lymphoma or leukaemia. Overall, 5.1% of the participants had a first-degree family history of lymphoma or leukaemia. Non-Hispanic white women, older women and women with higher BMI were more likely to have an affected first-degree relative. Family history did not differ according to socioeconomic status, smoking status or alcohol consumption.

A history of haematopoietic malignancy in any first-degree relative was associated with a significant increased risk of B-cell NHL (RR=1.52, 95% CI=1.11–2.07) (Table 2). Risk of B-cell NHL was significantly elevated for women who reported having a first-degree relative with NHL or Hodgkin's lymphoma (RR=1.74, 95% CI=1.16–2.60), but was not associated with having a first-degree relative diagnosed with a leukaemia (RR=1.35, 95% CI=0.87–2.09), when compared with women with no family history of any haematopoietic malignancy. Meanwhile, risks varied significantly by the gender of the affected family member. Women reporting a female relative with lymphoma had a 2.5-fold greater risk of B-cell NHL than those with no family history of any haematopoietic malignancy (RR=2.48, 95% CI=1.53–4.04), but those reporting a male relative with lymphoma were not at increased risk (RR=1.14, 95% CI=0.59–2.21). In contrast, an increased risk of B-cell NHL was found among women who had a first-degree male relative with leukaemia (RR=1.76, 95% CI=1.05–2.94), but not a female-affected relative (RR=0.95, 95% CI=0.45–2.01), compared with women with no family history of any haematopoietic malignancy.

The association with a parental-based family history of lymphoma or leukaemia was comparable with that of a sibling-based family history (results not shown). Similar to the associations based on gender, women having a lymphoma-affected mother had the elevated NHL risk (RR=2.92, 95% CI=1.60–5.31), as did those having a leukaemia-affected father (RR=2.17, 95% CI=0.1.19–3.95) (results not shown).

## Discussion

Our cohort study results confirm the evidence from population-based case–control studies and registry-based linkage studies of an increased NHL risk among individuals with a family history of haematopoietic malignancy (Pottern *et al*, 1991; Zhu *et al*, 1998; Chiu *et al*, 2004; Altieri *et al*, 2005; Chang *et al*, 2005; Goldin *et al*, 2005). The magnitudes of our RR estimates are closer to those from registry-based studies, in which family history data are verified, than to those from case–control studies, in which family history is generally self-reported and, therefore, subject to misclassification and recall bias.

We found that the association with a first-degree family history of haematopoietic malignancy varied by gender, being stronger for female relatives with a history of lymphoma and for male relatives with a history of leukaemia, as reported earlier from population-based registries (Czene *et al*, 2007). Among women in their study, those who had an affected female relative with haematopoietic malignancy had a higher RR of NHL than those with an affected male relative. Our study results differ from the InterLymph consortium results for women, a pooled analysis of 17 NHL case–control studies (11 population-, 6 hospital-based) (Wang *et al*, 2007). In InterLymph, RRs were higher when the family history involved lymphoma in a male, or leukaemia in a female, relative; response rates for participating studies varied, with some below 60% (cases) and some control rates below 50%. Moreover, RRs varied widely across individual studies in the InterLymph report. Therefore, selection bias in case–control studies, especially from a survival bias, is of particular concern if the pattern of NHL heritability is also associated with aggressiveness of NHL subtype or with NHL survival more generally. Although the InterLymph report provided risk estimates for major B-cell NHL subtypes (Wang *et al*, 2007), we were unable to evaluate such risks due to the small number of cases with a family history.

A limitation of our study is that our family history reports were self-reported. Concerns regarding the accuracy of self-reported family history of lymphoma were raised previously in a Swedish study (Chang *et al*, 2006), in which such a history was validated against population-based registries. In that study, the specificity of reporting haematopoietic malignancy was extremely high for both lymphoma cases and controls (98 and 99%, respectively), but the sensitivity was 60% for lymphoma cases and only 38% for controls. This resulted in a higher estimated RR of NHL associated with family history of haematopoietic malignancy when using self-reported data rather than registry-based data. Our self-reported family history probably reflects some degree of misclassification, but this is unlikely to be differential, and would tend to bias our RR estimate towards the null as our family history reports were obtained at study entry. Further, it is unlikely that the accuracy of self-reported data differed by gender of the affected relative.

As we asked about family history of lymphoma separately from that of leukaemia, it is possible that participants with a family history of CLL reported this as a leukaemia, not as a lymphoma, though CLL is part of the NHL case definition (Jaffe *et al*, 2001). When we excluded women with CLL from our case group, censoring them on their dates of diagnosis, we obtained essentially the same results as shown in Table 2 in which risk for NHL was associated with having a male relative with leukaemia.

A final limitation is that we did not ascertain cancer diagnoses among participants’ relatives during the follow-up period. Given that cohort members who developed NHL, compared with those who did not, would be more likely to have had additional familial diagnoses of haematopoietic malignancy, our RR estimates may underestimate the true association with first-degree family history.

In summary, we confirmed a positive association between family history of haematopoietic malignancy and NHL risk.

## Figures and Tables

**Table 1:** Selected baseline characteristics of 121 216 eligible women in the California Teachers Study in relation to family history of haematopoietic malignancy at baseline

		**Family history of lymphoma or leukaemia**		
	***N***	**No**	**Yes**	**Not known**
Total	121 216	113 225	6165	1826
				
*Age at cohort entry (years)*				
<35	12 741	10.7	4.0	18.2
35–44	21 448	17.9	13.5	19.2
45–54	36 468	30.1	29.3	30.8
55–64	23 475	19.2	23.3	15.3
65–74	17 797	14.5	19.1	11.5
75–84	9287	7.5	10.8	5.1
				
*Age-adjusted percentages*				
Race				
Non-Hispanic white	104 814	86.6	89.0	81.4
All other races/ethnicities	15 405	12.7	10.3	13.9
Unknown race/ethnicity	997	0.7	0.7	4.7
Socioeconomic status quartile rank				
1st (low)	5287	4.1	4.0	4.5
2nd	20 799	16.3	16.0	17.6
3rd	39 228	32.0	31.5	33.7
4th (high)	54 356	46.4	47.3	43.0
Unknown	1546	1.3	1.2	1.2
Smoking status				
Never	79 627	64.8	63.2	62.8
Former	34 684	29.4	31.2	29.2
Current	6181	5.2	5.2	7.6
Unknown	724	0.6	0.4	0.5
Alcohol consumption (g^−1^ day)				
None	38 619	31.3	31.8	30.4
<15	56 970	48.3	46.3	47.4
⩾15	19 301	16.1	17.3	17.4
Unknown	6326	4.3	4.6	4.8
Body mass index (kg m^−2^)				
<20	12 651	48.4	47.0	44.1
20–24.9	58 204	10.1	9.3	9.8
25–29.9	29 100	24.1	25.0	24.2
⩾30	16 414	14.1	15.4	17.6
Unknown	4847	3.3	3.3	4.3
Number of siblings				
0	11 878	10.1	8.3	0.2
1	35 560	30.4	28.0	0.1
2	30 147	25.6	24.7	—
3	18 147	15.3	16.1	—
4+	21 804	17.3	21.7	0.1
Adopted/unknown	3680	1.4	1.3	99.6

**Table 2:** Relative risk estimates and 95% confidence intervals for the association between family history of haematopoietic malignancy and B-cell NHL risk among women in the California Teachers Study

**Family history**	**Total person-years**	**Number of cases**	**RR (95% CI)**
None	1 053 996	432	1.00
			
*Lymphoma*	29 620	25	1.74 (1.16–2.60)
Male relative	16 572	9	1.14 (0.59–2.21)
Female relative	13 601	17	2.48 (1.53–4.04)
			
*Leukaemia*	29 211	21	1.35 (0.87–2.09)
Male relative	16 653	15	1.76 (1.05–2.94)
Female relative	12 960	7	0.95 (0.45–2.01)
			
*Any lymphoma or leukaemia*	57 153	44	1.52 (1.11–2.07)
Male relative	32 635	24	1.49 (0.99–2.25)
Female relative	26 037	22	1.58 (1.03–2.43)

Abbreviations: CI=confidence interval; NHL=non-Hodgkin's lymphoma; RR=relative risk.
